# Low-temperature methanation of fermentation gas with Ni-based catalysts in a multicomponent system

**DOI:** 10.1186/s13068-023-02455-4

**Published:** 2024-01-28

**Authors:** Jie Yin, Zihui Yao, Qizhi Zhao, Shikun Cheng, Xuemei Wang, Zifu Li

**Affiliations:** School of Energy and Environmental Engineering, Beijing Key Laboratory of Resource-Oriented Treatment of Industrial Pollutants, University of Science and Technology Beijing, Xueyuan Road No.30, Haidian District, Beijing, 100083 People’s Republic of China

**Keywords:** CO_2_ methanation, Ni–Fe/Al-Ti, catalyst, Low activation temperature, Fermentation gas

## Abstract

A large amount of greenhouse gases, such as carbon dioxide and methane, are released during the production process of bioethanol and biogas. Converting CO_2_ into methane is a promising way of capturing CO_2_ and generating high-value gas. At present, CO_2_ methanation technology is still in the early stage. It requires high temperature (300–400 ℃) and pressure (> 1 MPa), leading to high cost and energy consumption. In this study, a new catalyst, Ni–Fe/Al–Ti, was developed. Compared with the activity of the common Ni/Al_2_O_3_ catalyst, that of the new catalyst was increased by 1/3, and its activation temperature was reduced by 100℃. The selectivity of methane was increased to 99%. In the experiment using simulated fermentation gas, the catalyst showed good catalytic activity and durability at a low temperature and atmospheric pressure. Based on the characterization of catalysts and the study of reaction mechanisms, this article innovatively proposed a Ni–Fe/Al–Ti quaternary catalytic system. Catalytic process was realized through the synergism of Al–Ti composite support and Ni–Fe promotion. The oxygen vacancies on the surface of the composite carrier and the higher activity metals and alloys promoted by Fe accelerate the capture and reduction of CO_2_. Compared with the existing catalysts, the new Ni–Fe/Al–Ti catalyst can significantly improve the methanation efficiency and has great practical application potential.

## Introduction

Using biofuels is one of the most promising ways to replace fossil fuels [1]. Despite being a negative carbon energy source [2], bioethanol or biogas still have eco-unfriendly aspects in the production process. CO_2_, CH_4_, and other greenhouse gases are continuously released during fermentation and biomass residue treatment, leading to energy and carbon loss. The release of these greenhouse gases negatively impacts the environment [3–5]. In response to this problem, some scholars have recently proposed the technical path of converting CO_2_ in the mixture into methane through hydrogenation. In this process, CO_2_ emissions can be reduced, and waste gas can be converted into usable energy.

The technology of CO_2_ methanation originated from the French chemist Paul Sabatier in 1902 [6, 7]. He reported the hydrogenation methanation of carbon dioxide in the presence of the heterogeneous catalysis of transition metal, whose chemical reaction is presented below:

$${\text{4H}}_{{2}} \, + \,{\text{CO}}_{{2}} \, \to \,{\text{CH}}_{{4}} \, + \,{\text{2H}}_{{2}} {\text{O}}\quad \Delta {\text{H}}^{0} \, = \, - \,{165}\,{\text{KJ}}/{\text{mol}}$$

At present, methanation has some applications in coal, natural gas, ammonia, and hydrogen production industries, but it is not widely used in the new energy industries. The noble metal Ru was introduced into the Ni catalyst in biogas methanation in some researches [8]. At the reaction temperature of 300–400 ℃, the catalytic reaction shows good activity and obtains high-purity methane. Other studies [9, 10] on the influence of each component in the mixed gas on the catalytic reaction showed that in addition to the great toxicity of sulfur on the catalyst, the influence of other components, such as ammonia, methane and water, is very small. The above exploration provides a feasible basis for the application of methanation technology in this field.

The key to methanation technology is a catalyst [5, 7]. Among the many catalysts based on Ni, Ru, Fe, and Co, Ni has the most comprehensive advantages in efficiency and price. Thus, it has been studied and applied extensively [11–14]. However, Ni-based catalysts still have the disadvantages of easy deactivation and poor thermal stability. The biomass energy industry must meet energy-saving standards, low consumption, high efficiency, and safe operation. Thus, high requirements are proposed for catalysts.

At present, the research on the Ni-based catalytic system mainly focuses on investigating metal activity, selecting support, and adding additives. Previous studies [15–21] showed that introducing additives such as La, Ce, Co, or Fe into Ni catalysts can promote the dispersion of NiO and increase the amount of active nickel in the catalyst. This is due to the similar crystalline properties of its metal phase, which can easily dissolve into the lattice of Ni to change its dispersibility.

It has been also found that the formation of bimetallic alloy in the catalyst is an important reason to enhance the catalytic performance. Andersson [22] believed that that specific alloys can reduce the M–CO binding energy and result in higher CO methanation activities. Moreover, noble metals Ru, Rh, Pt and Pd can improve the reaction activity by increasing the reducibility of the primary Ni phase, by expanding the reaction pathway, or by raising the Ni dispersion [23].

In addition, three metals were combined to improve stability and catalytic activity. For example, the literature [20] showed that M*–Mn–Cu trimetallic catalysts can achieve nearly 99% of the CO_2_ conversion (M* refers to active metals).

The effect of support on the catalytic performance of the catalyst is not dominant. However, the strong interaction between metal and support considerably affects catalytic activity. Adding MgO or Cr_2_O_3_, La_2_O_3_, CeO_2_, ZrO_2_, TiO_2_ to common carriers can improve the interaction between active components and carriers, and the migration, agglomeration, and sintering of active components can be inhibited [24–28]. The study found metal ions in CeO_2_ and ZrO_2_ can also enter the lattice of the metal oxide supports, or formed a certain kind of segregated metal oxide phases supported on the support surface [27–33]. The composite carriers comprising TiO_2_, ZrO_2_, and other oxides are conducive to oxygen vacancy formation when the M^4+^/M^3+^ valence ratio is reduced; thus, CO_2_ adsorption capacity and catalytic activity can be improved [34–37].

The study on the surface methanation process of Ni–Co/ZrO_2_–CeO_2_ catalyst [12] showed that the reducibility and crystal structure of the catalyst improve with the enhancement of metal support interaction. These catalysts have nickel-active sites suitable for methanation, and composite oxides show good support and synergistic effects. It can be seen that the use of multiple active metals and composites is an effective way to improve the catalytic activity. There is a lot of research on Ni–Fe or Ni–Co bimetallic materials, but little study on the multiple relationship between bimetallic materials and composite carriers [19, 30–37]. In the present study, a new type of catalyst, Ni–Fe/Al–Ti, was developed, and the multielement composite catalytic system was explored to help it obtain high thermal stability, high selectivity, and high activity at a low temperature (< 250 ℃) and atmospheric pressure. Simulated experiments were conducted on the mixed gas after dehydration and desulfurization of fermentation gas to verify the aging resistance of the catalyst and realize the low-temperature methanation of fermentation gas and the efficient use of carbon.

## Materials and method

### Catalyst preparation

For catalyst support preparation [25, 38], γ-Al_2_O_3_ and TiO_2_ were separately calcined at 400 ℃ for 4 h. The first step in preparing Al–Ti composite supports was to dissolve the Al_2_O_3_ and TiO_2_ mixture with 1:1, 2:1, 3:1, and 4:1 ratios in diluted nitric acid. After being stirred for 30 min, the mixture was dried at 105 ℃, followed by calcination at 540 ℃ for 4 h. The composite support was denoted as mAl–nTi, where m and n represent the mass ratios of Al_2_O_3_ to TiO_2_, respectively, and Al–Ti refers to the composite supports in the present study.

All catalysts were prepared via wet impregnation method [39–41] at different loadings of 5%, 10%, 15%, 20%, and 25%. For Ni–Fe bimetallic catalysts, the mixture of Ni(NO_3_)_2_·6H_2_O (AR, 99.7%) and Fe(NO_3_)_2_·9H_2_O (AR, 99.7%) with 1:1, 2:1, 3:1, and 4:1 ratios was dissolved. The prepared support was added to the solution containing metal precursors. The mixture was stirred for 0.5 h prior to water bath oscillation and then dried at 120 ℃ for 24 h, followed by calcination at 480 ℃ for 4 h. The catalysts were denoted as xNi–yFe/support, where x and y refer to the mass ratios of Ni and Fe loaded on composite supports, respectively.

### Experimental setup

1. Experimental setup
  The CO_2_ methanation experiment was conducted at atmospheric pressure in a stainless steel fixed-bed tubular reactor (Fig. 1) with 4 mm diameter. A 0.5 g sample catalyst was retained between 20 and 40 mesh sieves. Before each reaction, the catalyst was reduced first at 540 ℃ for 60 min in H_2_. Then, the reaction gases mixed with CO_2_, CH_4_, and H_2_ were introduced into the reactor. The products were analyzed by gas chromatography-thermal conductivity detector (GC-TCD).

**Fig. 1:**
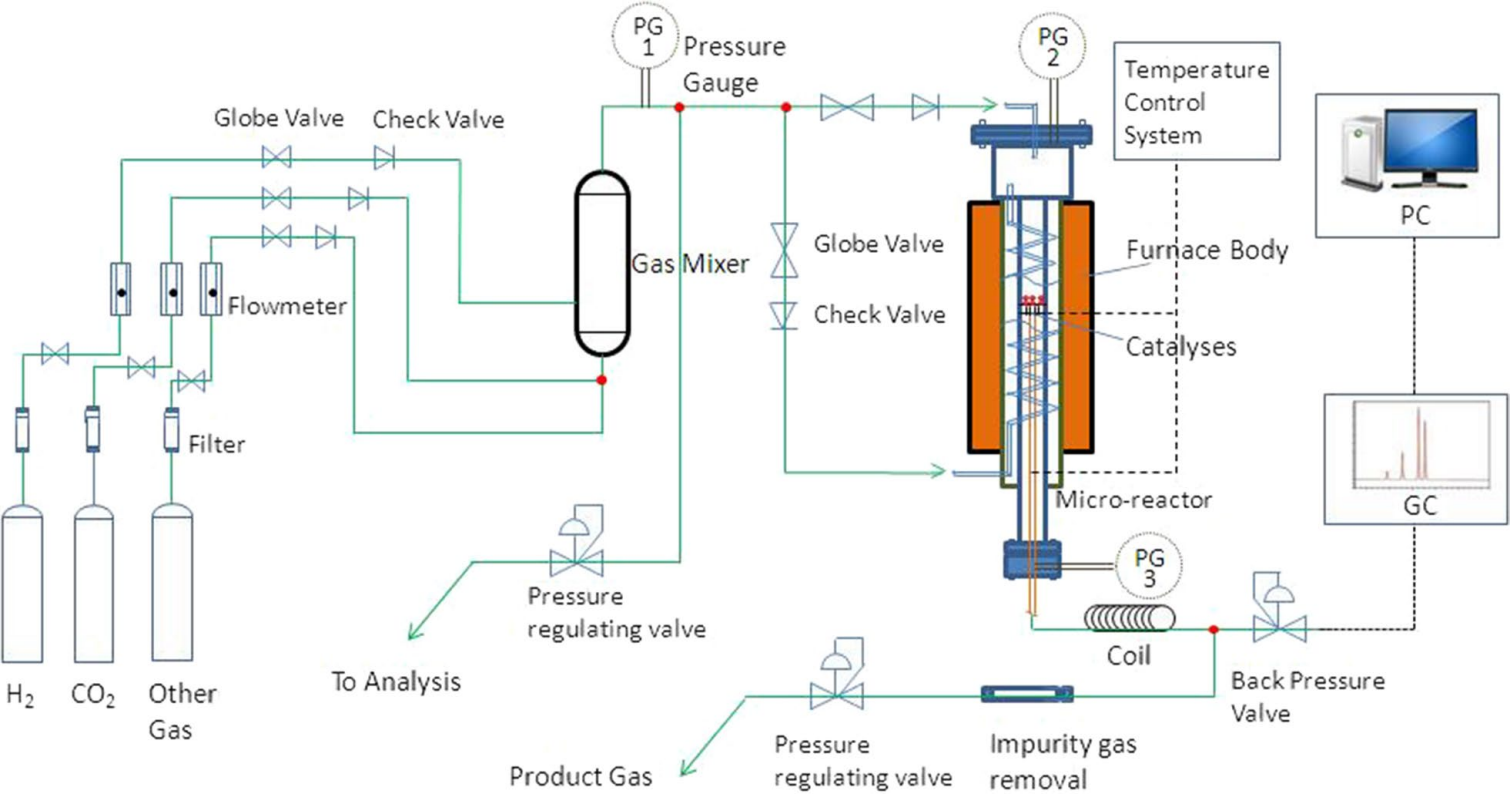
CO_2_ methanation reactor
2. Catalytic activity test

The catalytic activity was tested under a GHSV (gaseous hourly space velocity) of 3000 h^−1^ to 8000 h^−1^ at atmospheric pressure in the temperature ranging from 150 to 600 ℃. The reaction gases with a V(H_2_)/V(CO_2_) ratio of 4 were introduced into the reactor. The activity of the catalysts was evaluated via CO_2_ conversion and CH_4_ selectivity.

### Characterization analysis

The morphology of the catalysts was measured by a field emission scanning electron microscope (SEM; Hitachi, SU8100). The transmission electron microscope (TEM) images, high-resolution TEM (HRTEM) images, and energy dispersive X-ray spectroscopy (EDS) results were obtained by an ambient atmosphere spherical aberration correction electron microscope (Titan ETEM G2) at 300 kV.

The specific surface area of the catalyst was detected via vacuum method using an automated surface and pore size analyzer (NOVA1000, Quanta Chrome Instruments), with highly purified N_2_ as standard absorption gas at 77 K. The catalysts were pretreated by degassing at 250 ℃. The XRD patterns were detected on a Rigaku Ultima IV X-ray Diffractometer using Cu Kα radiation. The voltage and current of the parameter measurements were operated at 40 kV and 40 mA, with a scanning speed of 10°min^−1^.

X-ray photoelectron spectroscopy (XPS) was performed using an AXIS Supra X-ray photoelectron spectrometer with an Al K source (1486.8 eV, 12 mA, 20 kV). The binding energy of Ni 2p, Al 2p, Ti 2p, and O 1 s was calibrated with the C 1 s peak (BE = 284.8 eV) as a standard.

In situ FTIR measurements were performed to identify the adsorbed reaction intermediates in the CO_2_ methanation reaction. A Bio-Rad Digilab FTS-60A system equipped with a DTGS detector was used. Each spectrum was recorded at 4 cm^−1^ resolution, averaged over 64 scans. The spectra were measured under the stream of 4% H_2_, 1% CO_2_, and 95% He mixture (100 mL min^−1^) at 150, 200, 300, 400, and 500 ℃. H_2_ temperature-programmed reduction (H_2_-TPR) measurements were used in the present study to test the dispersion of the active metal on the surface of catalysts and the interaction of supports. A 0.1 g sample for each test was dehydrated with 40 mL/min Ar at 450 ℃. Afterward, the temperature was reduced to 50 ℃ before the introduction of a 5 vol% H_2_–95 vol% Ar mixture. Then, the temperature increased to 650 ℃ at a heating rate of 10℃/min. A TCD recorded the peak values.

CO_2_ temperature-programmed desorption (CO_2_-TPD) experiments were carried out in a fixed-bed reactor. A pulsed CO_2_ chemisorption was conducted at room temperature by injection of 0.50 mL of 15 mol% CO_2_ balanced with He in He stream. TPD was performed using He at a flow rate of 30 mL/min in the temperature range 40–900 ℃ at a heating rate of 10℃/min. The products were analyzed by a thermal conductivity detector (TCD).

(3) CO_2_ conversion and CH_4_ selectivity
  The methane concentration was determined with a TCD detector for GC by measuring its peak area. The CO_2_ conversion and CH_4_ selectivity were calculated using the following equations:
  1 $$X_{{{\text{CO}}_{2} }} (\% ) = \left[ {1 - \frac{{(CO_{2} )}}{{(CH_{4} ) + (CO_{2} )}}} \right] \times 100,$$
  2 $$S_{{{\text{CH}}_{{4}} }} (\% ) = \frac{{(CH_{4} )}}{{(CO_{2} )_{{}}^{0} - (CO_{2} )}} \times 100,$$
  where $(CO_{{2}} )^{0}$ refers to the initial CO_2_ concentration in the feeding gas; and $(CH_{{4}} )$ and $(CO_{{2}} )$ represent the methane and CO_2_ concentration in gas production, respectively.

## Results and discussion

### Catalyst characterization

#### SEM

**Fig. 2:**
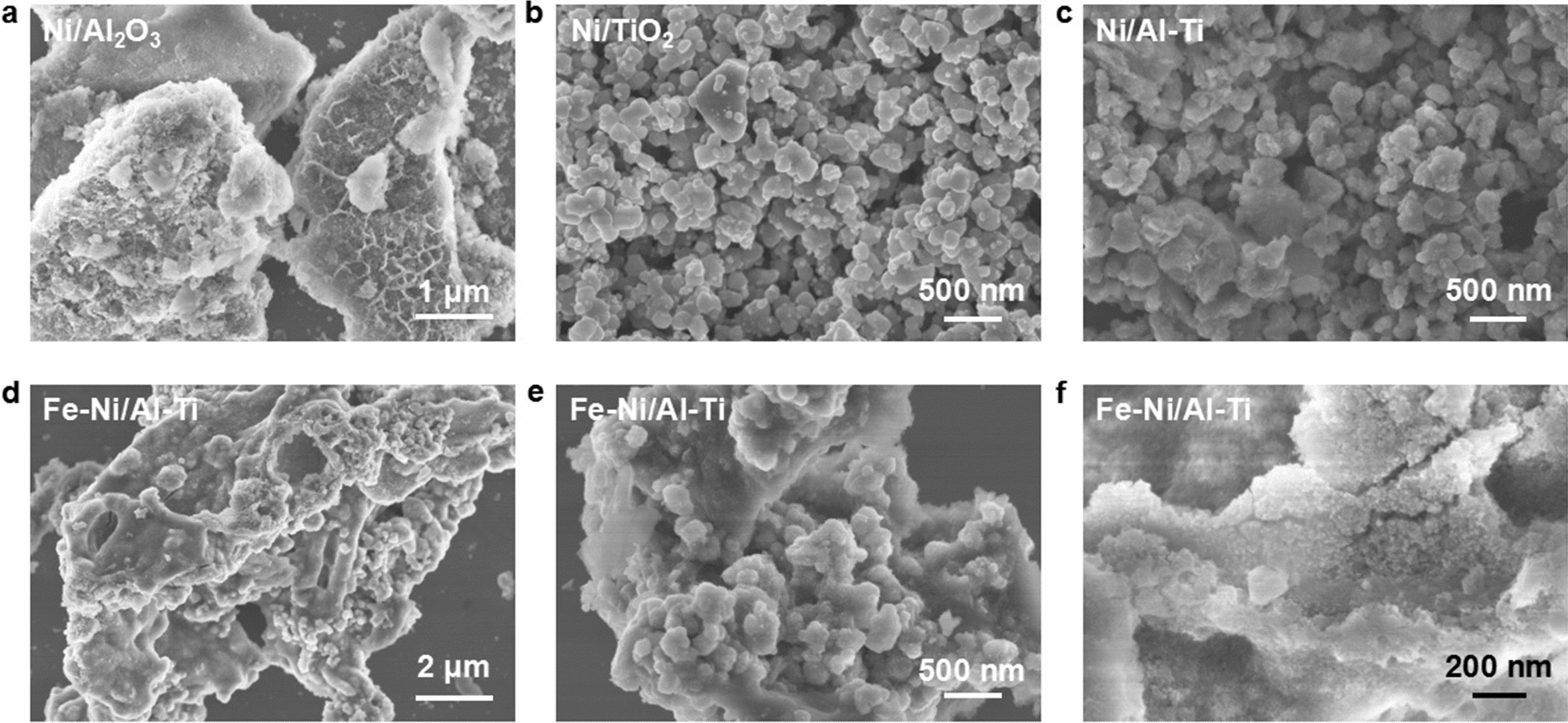
SEM images of different catalysts. **a** refers to Ni/Al_2_O_3_, **b** refers to Ni/TiO_2_, **c**–**f** refer to Ni-Fe/Al-Ti

The SEM images were taken to study the surface morphology of the catalyst. As shown in Fig. 2a, the surface of Ni/Al_2_O_3_ catalyst particles is loose and porous. The surface of Ni/TiO_2_ (Fig. 2b) particles is smooth without agglomeration, whereas Ni/Al–Ti shows aggregation (Fig. 2c). Moreover, 3Ni–Fe/2Al–Ti * (Fig. 2d, e) comprises many small particles of 100–150 nm. Using SEM (Fig. 2f) at a high power shows that the particle surface is loose and porous, with obvious holes. This observation confirms that mesopores exist in the surface material.

The particle size and morphology indicate that 3Ni–Fe/2Al–Ti has the morphology characteristics of the Al-based catalyst and Ti-based catalyst.

#### TEM analysis

**Fig. 3:**
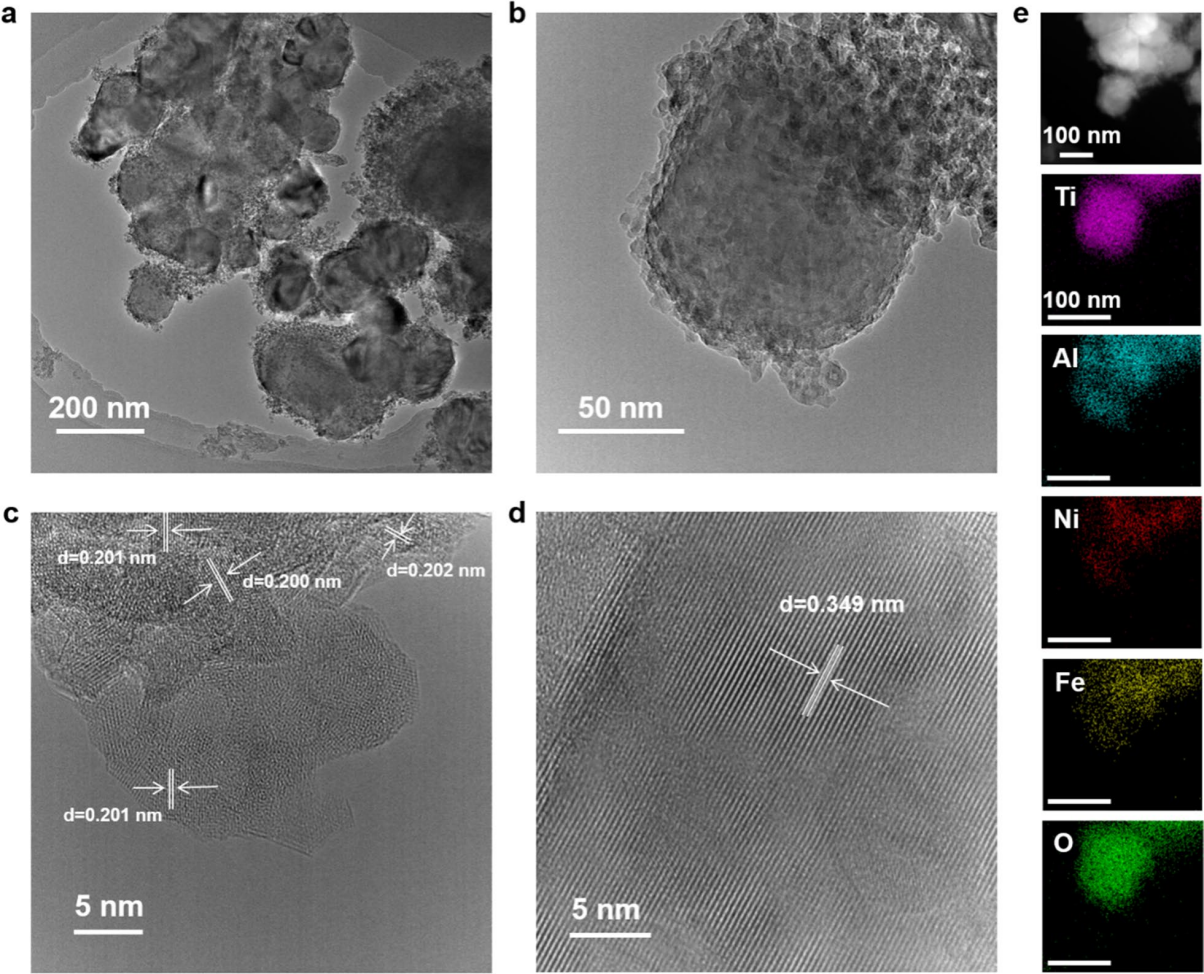
TEM photos of 3Ni–Fe/2Al–Ti. **a** and **b** refer to typical TEM images, **c** and **d** refer to HRTEM images, **e** refers to dark field images and EDS mappings

A TEM can be used to study the internal structural characteristics of materials. As shown in Fig. 3a, b, 3Ni–Fe/2Al–Ti comprises spherical particles with a diameter of 100–150 nm, and the outer surface is coated by the particles with a diameter of 5–10 nm. The HRTEM (Fig. 3c, d) image of 3Ni–Fe/Al–Ti shows that the crystallinity of the coating on the catalyst surface is poor, with the presence of a large number of nanoparticles with a lattice spacing measured to be 0.201 nm, which corresponds to the (111) crystalline surface of the Ni_3_Fe alloy. In addition, the spherical substrate particles showed obvious lattice streaks with a lattice spacing of 0.349 nm, which corresponds to the lattice type of anatase TiO_2_ [27]. The dark field image and EDS mapping (Fig. 3e) show the uniform distribution of Ti, Al, Ni, Fe, and O in the whole Fe–Ni/Al–Ti surface, revealing the uniform doping of Ni and Fe.

#### BET analysis

**Fig. 4:**
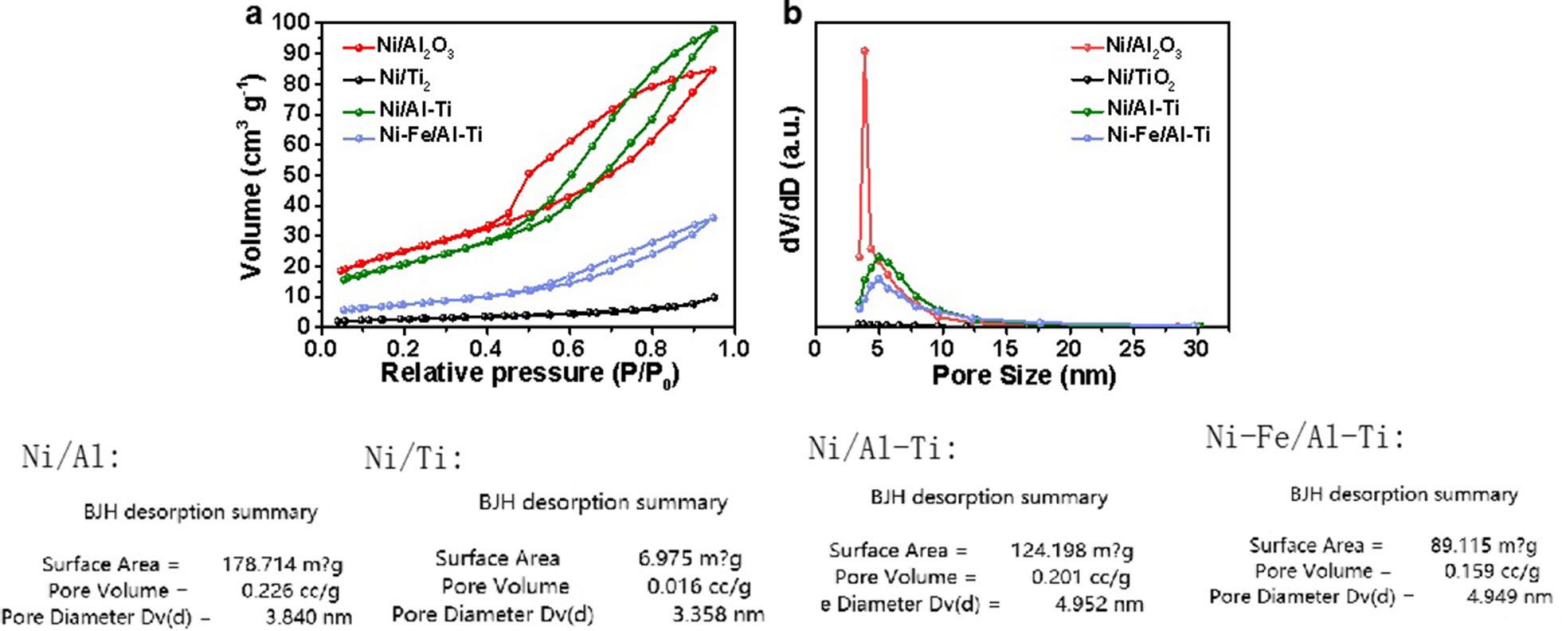
BET of different catalysts. **a** refers to N_2_ adsorption-desorption curves, **b** refers to BJH curves of pore size distributions

In general, catalysts with large specific surface areas and rich mesoporous structures can expose numerous active sites and induce material transport to improve catalytic efficiency [15, 16]. Figure 4 shows the N_2_ adsorption–desorption curves and Barrett–Joyner–Halenda (BJH) curves of different catalyst pore size distributions. Typical H3 hysteresis loops exist in the adsorption and desorption curves of Ni/Al–Ti and Ni–Fe/Al–Ti, representing the existence of mesoporous structure in the material. According to the BJH pore size distribution, the pore size of 3Ni–Fe/2Al–Ti is about 4.5 nm, which is greater than 3.8 nm of Ni/Al_2_O_3_, indicating that Al–Ti-based catalyst is more conducive to gas transmission. The BET specific surface area of Ni–Fe/Al–Ti shown in Table 1 is 76.7 m^2^/g, which is higher than that of Ni/TiO_2_ catalyst (7.4 m^2^/g) but lower than that of Ni/Al_2_O_3_ (179.7 m^2^/g). It can be seen that a larger specific surface area is not necessarily better. Appropriate specific surface area and abundant mesoporous structure are conducive to the diffusion of gas molecules and promote the catalytic reaction [42, 43].

**Table 1:** Specific surface area, pore volume, and pore size of catalysts

Item	Catalysts			
	Ni/TiO_2_	Ni/Al_2_O_3_	Ni/Al–Ti^*^	Ni–Fe/Al–Ti
Specific surface area (m^2^/g)	7.4	179.7	72.6	76.7
Pore volume (cm^3^/g)	0.02	0.30	0.17	0.15
Pore size (nm)	31.2	3.8	4.7	4.5

^*^Al–Ti refers to Al–Ti composite support

#### XRD

**Fig. 5:**
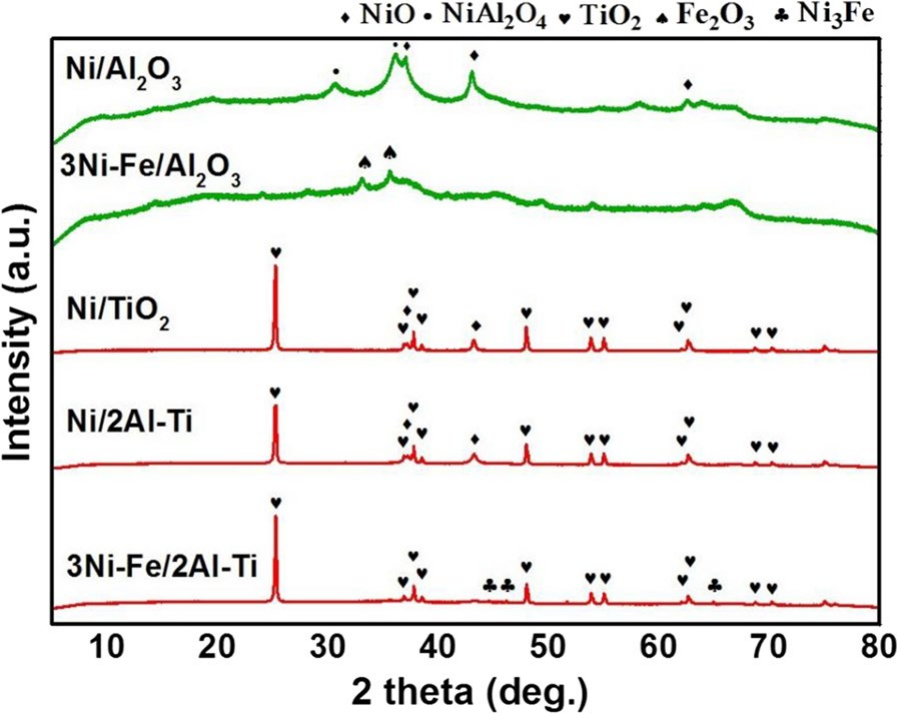
XRD patterns of catalysts

Figure 5 shows the XRD pattern of the catalyst. The peak passivation of Ni/Al_2_O_3_ and Ni–Fe/Al_2_O_3_ is amorphous. The difference is that the weak characteristic peak of NiO (PDF# 47–1049) appears in the Ni/Al_2_O_3_ spectrum (2θ = 44.5°, 51.8°, 76.3°) [34], whereas the characteristic peak of NiO in Ni–Fe/Al_2_O_3_ disappears. Previous studies [12, 13] confirmed that this finding is related to the high dispersion of NiO and that Fe doping increases the uniformity of Ni distribution.

The peak patterns of Ni–Fe/Al–Ti and those of Ni/Al–Ti and Ni/TiO_2_ are very similar to anatase (2θ = 25.3°, 37.8°, and 48° are anatase characteristic peaks), indicating that the catalysts have large amounts of TiO_2_ (PDF# 21–1272) crystals in the catalysts. Compared with the characteristic peaks of NiO in the Ni/Al–Ti and Ni/TiO_2_ spectra (2θ = 43.3° and 62.9°), that in the Ni–Fe/Al–Ti spectrum has no NiO; however, weak characteristic diffraction peaks of Ni–Fe alloy are observed at 44.611°, 46.168°, and 64.957° [30, 37]. This finding indicates that some forms of Ni–Fe alloy may also be formed in the 3Ni–Fe/2Al–Ti catalyst. Moreover, in 3Ni–Fe/2Al–Ti, there is no NiAl_2_O_4_ peak (PDF# 10–0339) in Ni/Al_2_O_3_ spectrum, indicating that nickel aluminate is well inhibited.

#### XPS

**Fig. 6:**
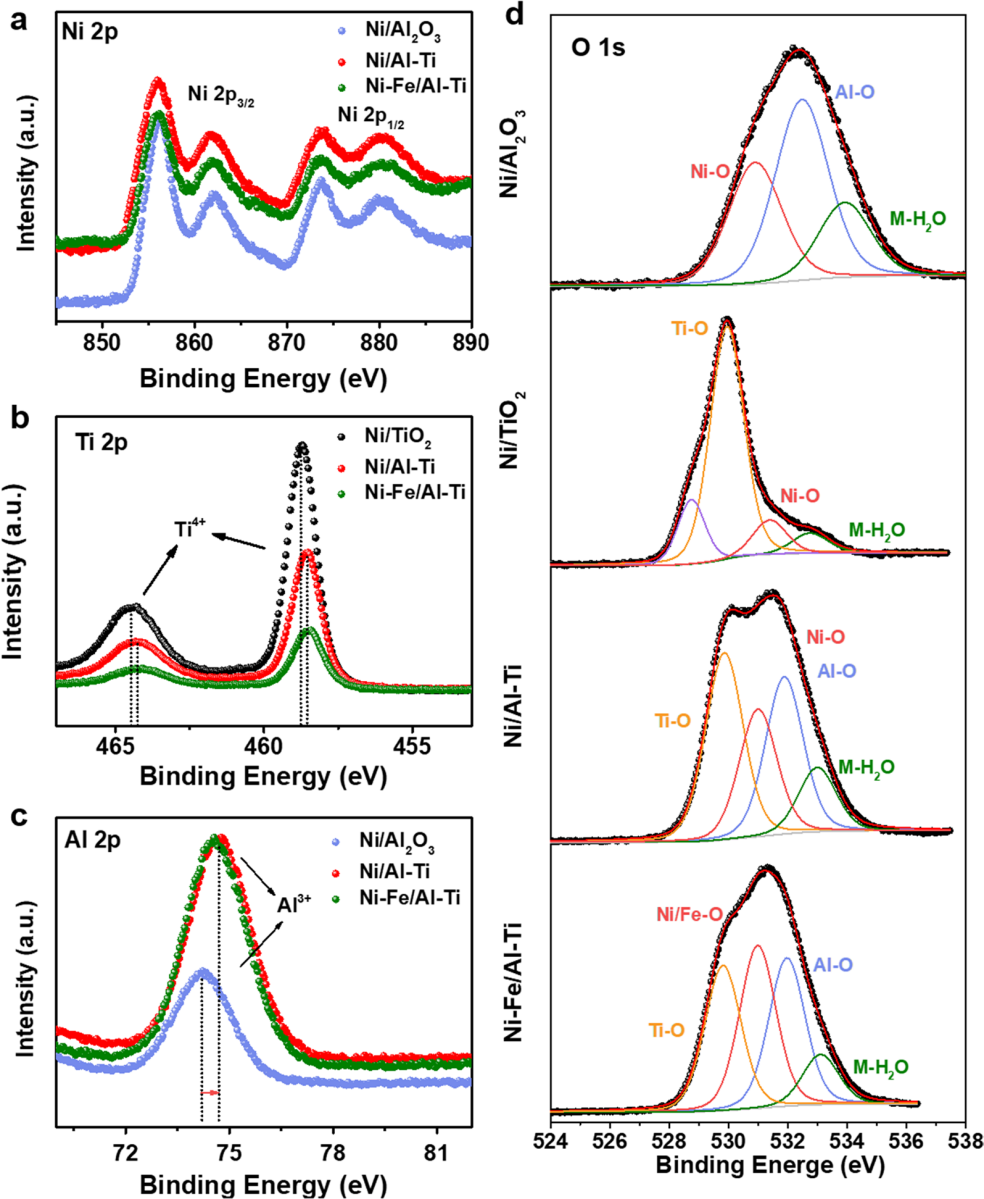
XPS profiles of catalysts. **a** refers to Ni 2P spectrum, **b** refers to Ti 2P spectrum, **c** refers to Al 2P spectrum, **d** refers to O 1s spectrum

Figure 6 reveals the XPS detection spectrum of the reduced catalyst. In the fine spectrum of Ni2P (Fig. 6a), four groups of peaks are found at the binding energies of 856.5, 861.7, 873.8, and 881.8 eV, which are attributed to Ni2p3/2 (NiO), Ni2p3/2sat (NiO), Ni2p1/2sat (NiO), and Ni2p1/2sat (NiO), respectively [44–46].

The fine spectra of Ni2p display four sets of the peaks at the binding energies of 856.5, 861.7, 873.8, and 881.8 eV, which can be assigned to Ni2p3/2(NiO), Ni2p3/2sat(NiO)), Ni2p1/2sat(NiO), and Ni2p1/2sat(NiO), respectively [25]. This result suggests that in the catalyst, Ni primarily exists in the form of NiO.

The fine spectrum of Ti2P (Fig. 6b) reveals that Ni/TiO_2_ has obvious double peaks at 458.7 and 464.5 eV attributed to Ti^4+^ [47, 48]. By comparison, the binding energy of Ti in the Al–Ti composite catalyst moves in the negative direction. According to the literature [27, 45], it may be caused by the Ti–OH formation or the Ti–O bond fracture, and this change can be reduced easily in the catalytic reaction to form an oxygen vacancy on the Ti bond on the catalyst surface. Figure 6c (Al2p spectrum) shows that the single peak of Ni/Al_2_O_3_ catalyst at 74.2 eV is due to the characteristic peak of Al_2_O_3_ [49]. In contrast, the Al peak of 3Ni–Fe/2Al–Ti increases from 74.23 eV to 74.56 eV and the Ti peak decreases from 458.79 eV to 458.55 eV, which may be due to the result of electron transfer. The spectrum of Ni/Al_2_O_3_ can fit Al–O bond and Ni–O bond at 532.3 eV and 530.9 eV, which proves that O is coordinated with Al and Ni, respectively [34, 38]. The spectra of Ni/TiO_2_ were fitted to Ti–O bond and Ni–O bond at 529.9 eV and 531.1 eV, respectively. The three peaks of Ni/Al–Ti and 3Ni–Fe/Al–Ti spectra are also due to Ti–O, Al–O and Ni–O bonds, respectively.

#### *H*_*2*_*-TPR*

**Fig. 7:**
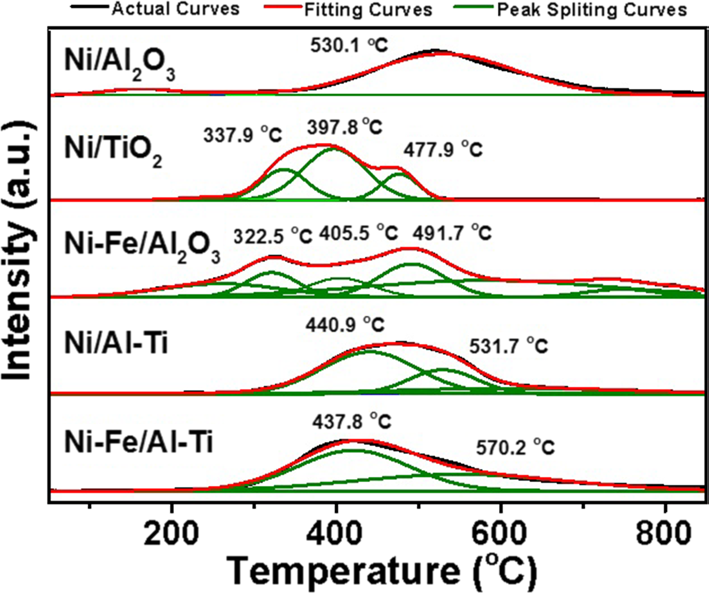
H_2_-TPR profiles of catalysts

The H_2_-TPR profiles can characterize the reducibility of catalysts and the interaction between metal and support. The H_2_-TPR spectrum of the prepared catalyst is shown in Fig. 7. Compared with the main reduction peak of Ni/Al_2_O_3_, that of 3Ni–Fe/2Al–Ti is advanced from 530℃ to 437℃, and the peak area increases to 22.2, an increase of 44%.

Comparing the H_2_-TPR patterns between Ni–Fe/Al_2_O_3_ and Ni/Al_2_O_3_ catalysts shows that the first peak shifts forward to 322.5 ℃ because of the addition of Fe [24]. The reduction peak can be attributed to Fe_2_O_3_ reduction, and the main peak at 491.7 ℃ represents the NiO reduction [13]. The distance between the two peaks indicates that the reduction reactions for Fe and Ni occur in different temperature ranges. The spectrum of 3Ni–Fe/2Al–Ti shows a single wide peak, which reveals the combination of the two peaks. This finding indicates that Ni and Fe may synergistically affect the Al–Ti support.

The effect of Ti can be observed by comparing Ni/Al–Ti and Ni/Al_2_O_3_. The main hydrogen consumption peak of the former is advanced to around 90℃, and the peak area is increased by more than 34%. The possible reason is that the reduction of Ti–OH or Ti–O on the catalyst surface increases hydrogen consumption. Many oxygen vacancies of Ti bonds appear on the catalyst surface after deoxidation, thereby helping improve the catalytic activity. Similar conclusions can be confirmed by other studies [27, 35].

#### *CO*_*2*_*-TPD*

**Fig. 8:**
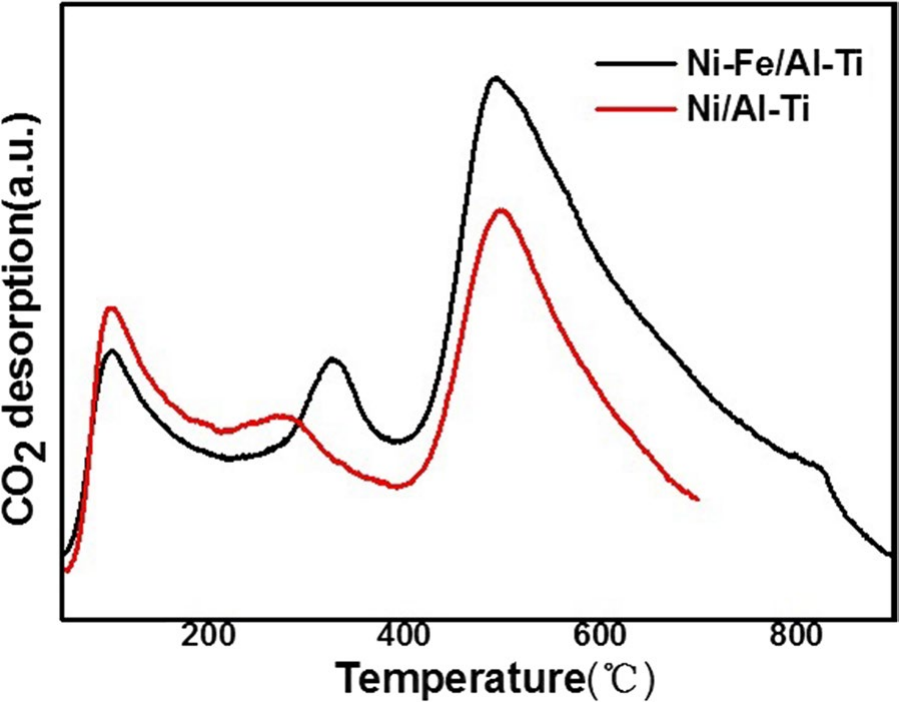
CO_2_-TPD profiles of catalysts

Figure 8 shows the CO_2_-TPD spectra of catalysts Ni/Al–Ti and Ni–Fe/Al–Ti. From the graph, it can be seen that both catalysts have three desorption peaks, and three different CO_2_ desorption peaks were observed in both catalysts. The low-temperature desorption peak (< 200 ℃) is attributed to weakly interacting CO_2_ molecules with weak alkaline sites on the catalyst surface [36]. The peak between 200 and 400 ℃ belongs to the desorption of CO_2_ molecules with moderate interactions with moderate alkaline sites.

The high temperature peak (> 400 ℃) belongs to strongly interacting CO_2_ species, corresponding to the presence of strongly alkaline sites [28]. From the comparison of the two catalysts, it can be seen that the low temperature peak (99 ℃) and high temperature peak (495 ℃ and 499 ℃, respectively) of both catalysts appear at the same position. However, the mid-temperature peak of Ni–Fe/Al–Ti shifts towards the high temperature direction, and the peak area increases, indicating that the bimetallic catalyst has an enhanced adsorption capacity for CO_2_ [24]. Combining XRD and XPS characterization, it is speculated that the addition of Fe changes the electronic effect on the catalyst surface, making CO_2_ more easily adsorbed.

#### In situ* FTIR measurements*

**Fig. 9:**
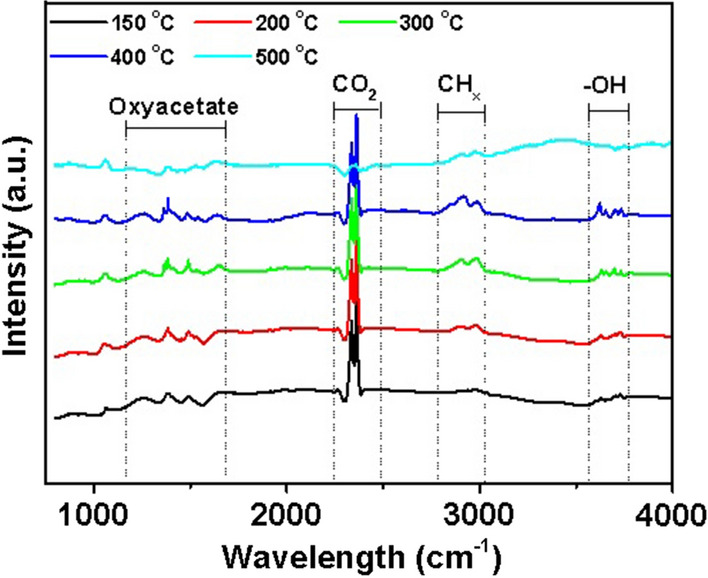
In situ IR spectra of coadsorption of CO_2_ and H_2_ at different temperatures

Figure 9 shows the in situ IR spectra of the coadsorption of CO_2_ and H_2_ on the Ni–Fe/Al–Ti catalyst at different temperatures. In addition to the relatively strong CO_2_ adsorption peak, three adsorption peak regions exist. The 1700–1200 cm^−1^ region belongs to the oxygen acid salt species [46, 47]. At 150 ℃, the absorption peak belongs to the formate species (1378 cm^−1^), and two absorption peaks belong to the carbonate species (1251 and 1489 cm^−1^). As the temperature increases to 200 ℃, the concentration of carbonate and formate species gradually increases. The absorption peaks at 1362 and 1493 cm^−1^, belonging to other formate species, appear when the temperature exceeds 300 ℃, whereas the absorption peak at 1658 cm^−1^ belongs to bicarbonate species [29]. Formate species reach the maximum at 400 ℃, whereas carbonate species decrease. When the temperature increases to 500 ℃, the absorption peak in this region weakens or even disappears. This finding shows that the formation of oxonate’s intermediate species is inhibited at high temperatures. The absorption peaks in the 3000–2900 cm^−1^ region belong to CHx species. They increase significantly at 200 ℃ and reach the highest at 300℃. The peak strength of methane decreases continuously with the increase in temperature. The 3750–3200 cm^−1^ region belongs to the hydroxyl group, and the related species may be water, alcohol, and carboxylic acids [47, 48]. The peak intensity reaches the maximum at 400℃ and decreases gradually while the temperature continues to increase. The above analysis shows that no CO species exists in the reaction. Therefore, carbonates, formates, or other oxygen-containing salts are most likely intermediate species.

### Catalytic activity

#### Catalytic activity and selectivity at different temperatures

**Fig. 10:**
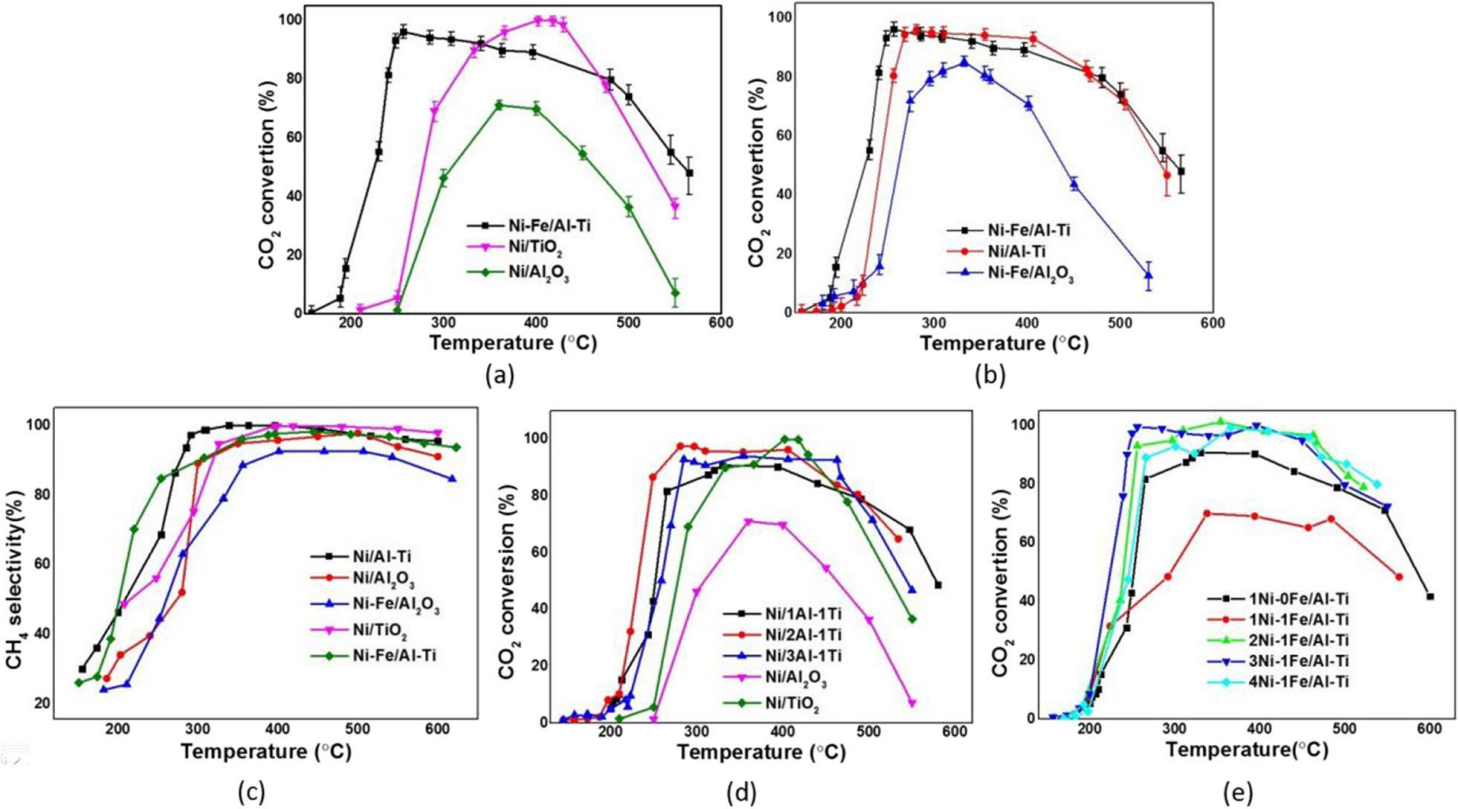
Effects of reaction temperature on CO_2_ conversion and CH_4_ selectivity for catalysts. **a** refers to effects of reaction temperature on CO_2_ conversion for Ni-Fe/Al-Ti, Ni/TiO_2_ and Ni/Al_2_O_3_, **b** refers to effects of reaction temperature on CO_2_ conversion for Ni-Fe/Al-Ti, Ni/Al-Ti and Ni-Fe/Al_2_O_3_, **c** refers to effect of temperatures on CH_4_ selectivity for catalysts, **d** refers to activity of composite supported catalysts with different Ni loadings, **e** refers to effects of Ni/Fe ratios on activity catalyst loading on composite supports

Figure 10a shows the catalyst’s CO_2_ conversion rate at different temperatures. The activation temperature (T50 referring to the temperature when the CO_2_ conversion is 1/2 of the peak) and the maximum activity temperature (Tpeak, referring to the temperature when the CO_2_ conversion is the peak) of Ni–Fe/Al–Ti are 210 and 256 ℃, respectively, which are approximately 90 and 104 ℃ lower than the T50 and Tpeak of Ni/Al, respectively. This finding indicates that the activity of the new catalyst is greatly improved.

Figure 10b shows that the maximum conversion rate of 3Ni–Fe/Al–Ti is 33% higher than that of Ni–Fe/Al_2_O_3_, revealing that the Al–Ti composite support is conducive to improving the CO_2_ conversion rate of the catalyst. The comparison between Ni–Fe/Al (255 and 332 ℃) and Ni/Al_2_O_3_ (300 ℃ and 400 ℃) shows that introducing Fe can effectively reduce the reaction temperature during catalysis.

Although the maximum conversion rate of Ni/TiO_2_ (99.9%) is the highest, its Tpeak (418 ℃) is too high. Ni–Fe/Al–Ti (98.5%) has high conversion, and its T50 and Tpeak are the lowest, up to 210 ℃ and 256 ℃. Therefore, Ni–Fe/Al–Ti catalyst shows the best comprehensive performance in both CO_2_ conversion efficiency and low-temperature-conversion performance. In some literatures [22–28], the optimal temperature for methanation is typically between 300 and 400 ℃. And when the catalytic temperatures are below 250 ℃, the conversion of carbon dioxide is less than 60%. Thus, it shows that Ni–Fe/Al–Ti has obvious advantages in high activity at low temperature.

Figure 10c shows the CH_4_ selectivity of the catalyst at different temperatures. The maximum selectivity ranking is Ni/TiO_2_ > Ni/Al–Ti > 3Ni–Fe/Al–Ti > Ni–Fe/Al > Ni/Al, and the maximum selectivity of Ni/TiO_2_ can reach 99%.

At < 280 ℃, the CH_4_ selectivity values of Ni/Al_2_O_3_, Ni/TiO_2_, and 3Ni–Fe/Al_2_O_3_ are less than 75%, indicating their poor CH_4_ selectivity values in low temperatures. By contrast, 3Ni–Fe/Al–Ti (91%) and Ni/Al–Ti (93%) catalysts have better CH_4_ selectivity than single-support catalysts at 280 ℃, indicating that Al–Ti composite support catalysts have advantages in low-temperature selectivity.

### Effect of promoters and supports on catalytic activity

Composite support catalysts with different Al/Ti ratios are prepared and compared with one-component support catalysts to study the effect of support components on catalyst activity.

As shown in Fig. 10d, compared with the catalytic temperatures of Ni/TiO_2_ and Ni/Al_2_O_3_, the catalytic temperature of the composite support catalyst is significantly reduced by 80–120 ℃, and the conversion rate of the composite support is greater than 85% in the 260–460 ℃ range. In particular, the composite support catalysts have good performances in low-temperature activity and high-temperature resistance and a suitable wide temperature range of catalysis.

The comparison of the catalysts with different Al/Ti ratios indicates that the best Al/Ti ratio is 2:1. The catalyst with a 2:1 Al/Ti ratio has the lowest T50 (223 ℃) and Tpeak (281 ℃), and its maximum conversion rate is 97%.

Figure 10e reveals that Fe promotion increases the catalyst activity at a low temperature. Compared with the T50 of Ni-based catalyst, that of 3Ni–Fe/Al–Ti is reduced by 60–80 ℃. The optimal activity of > 98% selectivity is obtained when the Ni/Fe ratio is 3:1, with T50 of 206 ℃ and Tpeak of 250 ℃. When we discuss the catalytic activity of different proportions of metals, we can observe some interesting trends. At approximately 250 ℃, the CO_2_ conversion rates of 1Ni–0Fe and 1Ni–1Fe catalysts are below 50%, while the CO_2_ conversion rate of the 2Ni–Fe catalyst reaches 78%, which is higher than 53% of the 4Ni–Fe catalyst. They are all much lower than 98% of 3Ni–Fe. It indicates that an appropriate amount of Fe (Ni/Fe ratio of 3:1) can greatly improve catalytic activity, while a small and excessive amount of iron can reduce the effectiveness of activity enhancement. When the temperature reaches 300 ℃, the conversion rates of 2Ni–Fe, 3Ni–Fe, and 4Ni–Fe catalysts all exceeds 90%, and the differences among the three become negligible. It can be seen that an appropriate amount of Fe has a significant effect on activity enhancement, especially at low temperatures. But the effect of Fe on catalytic activity is no longer significant in the high temperature. Taking into account low-temperature activity, high-temperature activity and methane selectivity, 3Ni–Fe/2Al–Fe catalyst is the optimal option (Table 2).

**Table 2:** Performances of Ni–Fe/Al–Ti and Ni/Al_2_O_3_ catalysts

Catalyst	Specific surface area (m^2^/g)	Pore size (nm)	Apparent activation energy (KJ/mol)	Temperature for activity initiation (℃)	Temperature for highest activity (℃)	Reaction rates (mol CO_2_·g Ni^−1^·h^−1^)	CO_2_ conversion (%)	CH_4_ selectivity (%)
Ni/Al_2_O_3_	179.7	3.8	177.5	300	360	0.51	71	100
3Ni–Fe/2Al–Ti	76.7	4.5	98.0	206	254	0.38	95	99

### Different reaction conditions on catalytic activity

**Fig. 11:**
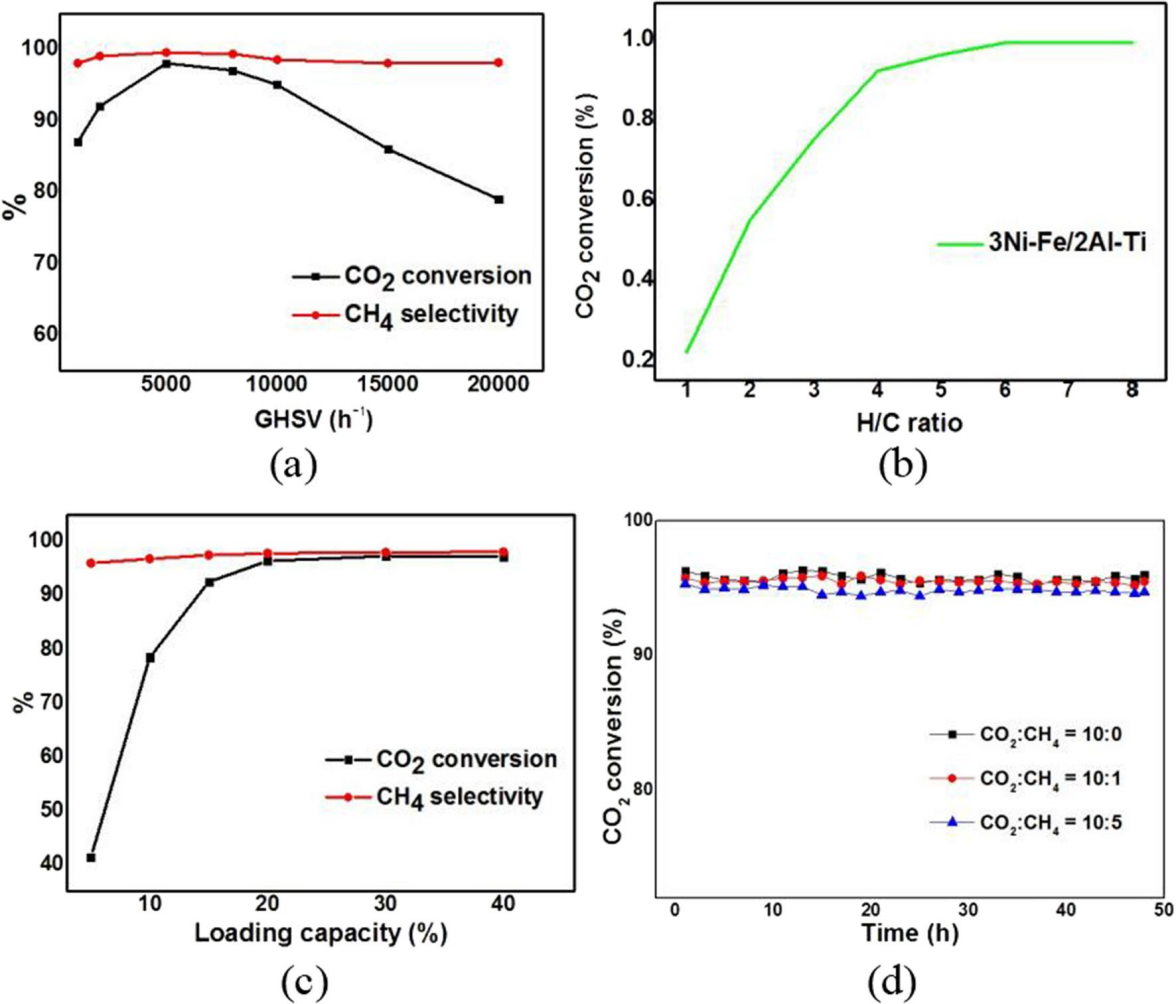
Effects of reaction conditions on CO_2_ conversion and CH_4_ selectivity for catalysts. **a** refers to effects of GHSV on catalysts, **b** refers to effect of H/C ratio on catalysts, **c** refers to effect of loading capacity on catalysts, **d** refers to effect of different mixture ratios of CH_4_ and CO_2_ gas as simulated tail gas of upgraded biogas on reaction stability

The catalytic performance of 3Ni–Fe/2Al–Ti catalyst was investigated under different process conditions. From Fig. 11a, it can be seen that space velocity has a significant impact on the CO_2_ conversion rate in catalytic reactions. When the airspeed ranges from 2000 to 10000 h^−1^, there is a process of first increasing and then decreasing the CO_2_ conversion rate. This is because the heat released by the reaction per unit time at low airspeed cannot be discharged in a timely manner, which affects the catalytic activity of the catalyst [24]. At higher airspeed, the number of reaction molecules per unit time increases and more heat is released. When the airspeed continues to increase (> 10000 h^−1^), the contact time between the feed gas and the active components of the catalyst decreases, resulting in a decrease in conversion rate due to insufficient reaction. The methane conversion rate reaches its maximum value at GHSV of 6000–8000 h^−1^. During the entire process, the catalyst exhibited good methane selectivity (> 95%), and changes in space velocity had no significant impact on methane selectivity.

The ratio of H_2_ to CO_2_ in the feed gas also has a certain impact on the conversion rate and product distribution of CO_2_. From Fig. 11b, it can be seen that 3Ni–Fe/2Al–Ti, at a temperature of 300℃, transforms almost 100% CO_2_ into CH_4_ when the H_2_ to CO_2_ flow rate ratio increases to 6, reflecting the high activity of the catalyst. Taking into account factors such as yield and economy, the optimal hydrogen carbon ratio V (H_2_)/V (CO_2_) = 4 should be adopted.

To investigate the effect of metal loading on catalytic activity, 3Ni–Fe/2Al–Ti catalysts with metal loading ranging from 5 to 40% were prepared and their catalytic performance was investigated. From Fig. 11c, it can be seen that the CO_2_ conversion rate and CH_4_ selectivity both increase with the increase of metal loading, but the increasing trend slows down when the loading exceeds 15%. An increase in loading capacity within a certain range is beneficial for improving catalyst activity, but an excessive loading capacity may lead to the accumulation of free catalytic components on the surface of the support, and even high-temperature sintering phenomenon [28]. Overall, the optimal loading capacity for Ni in the experiment is 15%.

In the experiment using simulated fermentation gas and biogas as the feed gas, the catalytic stability of the Ni–Fe/Al–Ti catalyst is tested at 250℃. As shown in Fig. 11d, the Ni–Fe/Al–Ti catalyst always maintains approximately 96% CO_2_ conversion without attenuation in the atmosphere with different CO_2_ and CH_4_ ratios. This observation indicates that the catalyst has good durability.

In summary, the most outstanding performance of 3Ni–Fe/2Al–Ti is its ability to maintain a CO_2_ conversion rate of 98% at low temperature (250 ℃), in contrast to a rate of below 60% at the same temperature in other researches [15–18]. Furthermore, 3Ni–Fe/2Al–Ti can achieve the same reaction conditions under atmospheric pressure as other catalyst must under 1 MPa (a H/C ratio of 4:1, a metal loading of 15%, a GHSV of 8000 h^−1^ and a continuous 48 h CO_2_ conversion rate of 96%).

### Mechanism of Ni–Fe/Al–Ti catalyst in CO_2_ methanation

The methanation mechanism is still controversial. Many researchers unanimously believe that CO_2_ hydrogenation is closely related to active metals and supports, and different methane reaction pathways appear on the surface of catalysts with different components [24–27, 35].

The main body of Ni–Fe/Al–Ti before modification is Ni/Al_2_O_3_, and its mechanism indicates that the Al_2_O_3_ carrier provides a good loading surface for the Ni distribution. The catalytic reaction occurs on the surface of Ni metal particles, and the reaction path is shown as below: [25]

3 $${\text{H}}_{{2}} + {\text{CO}}_{{2}} + {\text{Ni}} \to {\text{NiHCOOH}} \to {\text{ONiCHOH}} \to {\text{HONiCH}}_{{3}} \to {\text{CH}}_{{4}}$$

The dissociation of hydrogen and the fracture of the C=O bond occur under the action of Ni. This process highly depends on Ni [34]. However, the phenomenon of simultaneous adsorption and desorption of CO_2_ may occur because of the weak adsorption of C and O by Ni and may lead to a low conversion rate [25].

Ni–Fe/Al–Ti is modified by adding Ti and auxiliary Fe to the Al carrier. The experimental results indicate that the activity increases, the activation temperature decreases, and the reaction rate increases. The underlying possible reasons include the following.

The Ni–Fe/Al–Ti catalyst has bimetallic active substances, and the Ni–Fe alloy is discovered via XRD. The XPS and H_2_-TPR analyses indicate that the composite carrier may have many oxygen vacancies. After studying the interaction between carbonate species and oxygen vacancies on the carrier surface, some researchers believe that CO_2_ adsorption sites are mainly oxygen vacancies rather than metal sites, and the intermediate product of CO_2_ hydrogenation adsorbed by oxygen vacancies is oxalate species. In the FTIR spectrum, many carbonate and formic acid species can be found. It appears to be more in line with Ashok et al.’s hypothesis [15, 18], rather than the pathway that produces CO intermediate species [49–51].

**Fig. 12:**
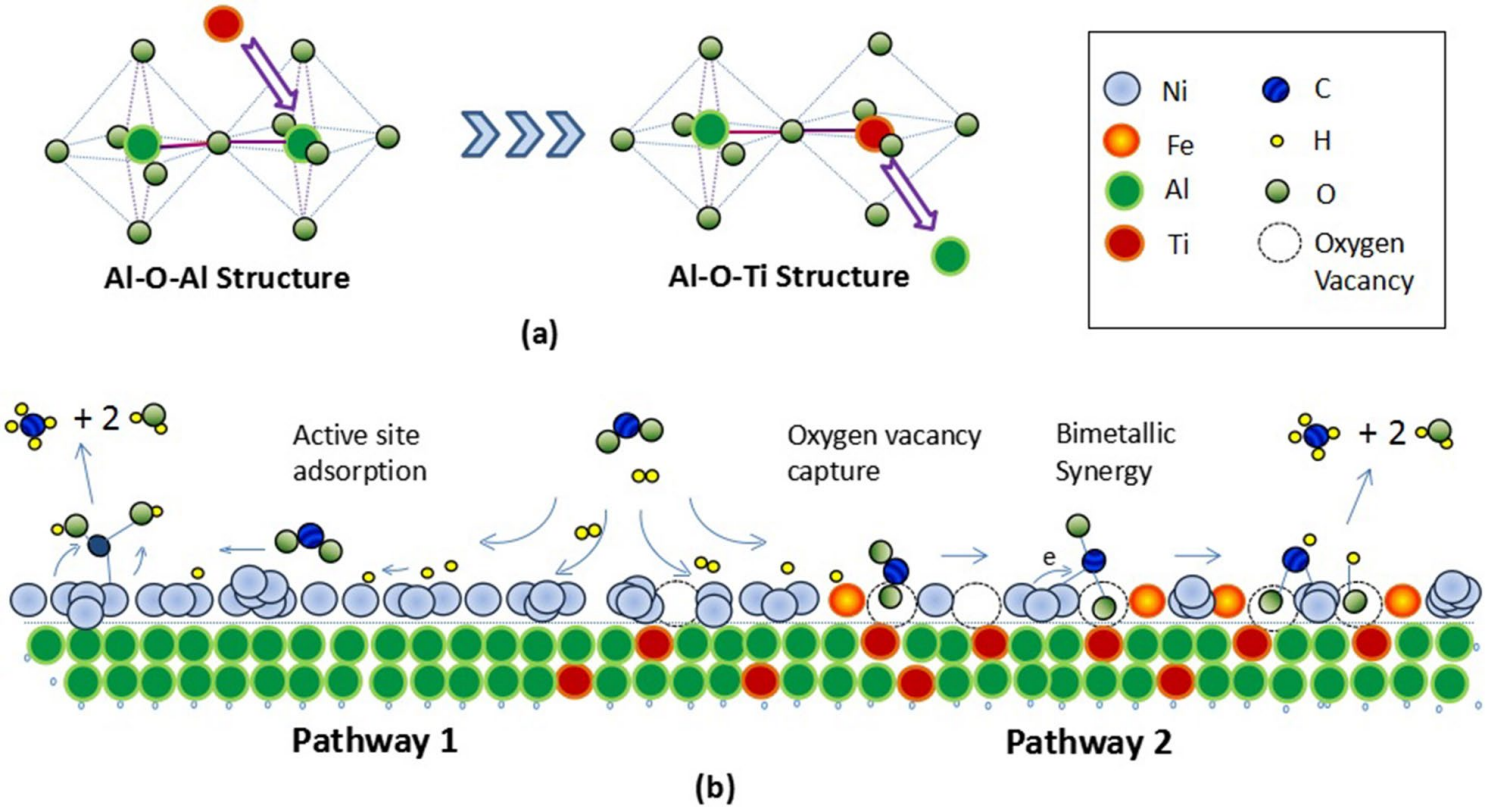
Mechanism map of catalysts in CO_2_ methanation. **a** refers to formation of Al-O-Ti structure of composite support, **b** refers to two reaction pathways of CO_2_ methanation on the catalysts

After CO_2_ adsorbs on the support, it is activated and transferred to the metal. This transfer process is determined by the interface between metal and support [44, 52]. The analysis of the carrier indicates that given the similar radii of Ti^4+^ and Al^3+^ ions in the composite carrier, they can enter each other’s lattice, as shown in Fig. 12. Thus, their original crystal morphology is broken, and specific Al–O–Ti chemical bonds are formed while preventing the generation of nickel aluminum spinel. The XRD analysis shows that the newly added Al–Ti characteristic peaks and nickel aluminum spinel characteristic peaks disappear. Undoubtedly, it improves the metal–carrier interface, which is crucial for enhancing the activity.

In summary, Ni–Fe/Al–Ti is a quaternary system with a complex reaction mechanism. Based on the literature, the main pathways are: H_2_ dissociation on Ni or Ni–Fe surfaces. CO_2_ is captured by oxygen vacancies on the surface of the composite carrier and then transferred to the metal or hydrogen via spillover to the support. CO_2_ reacts with hydroxyl groups to form carbonate and formate species, and hydrogenation continues until methane is finally formed. However, some chemical intermediates in CO_2_ methanation are difficult to measure, and further theoretical research is needed to determine their mechanisms.

## Conclusions

In this paper, a new quaternary system of catalyst is proposed and a new high-efficiency CO_2_ methanation catalyst 3Ni–Fe/2Al–Ti was prepared using impregnation and ultrasonic methods. The mesoporous structure and Ni–Fe bimetallic structure on the catalyst helped improve the catalytic activity and reduce the reaction temperature. Methane selectivity was increased to 99%, and the activation temperature was reduced to 206 ℃. Moreover, the reaction rate of 3Ni–Fe/2Al–Ti was 1.3 times that of Ni/Al_2_O_3_. Characterization and mechanism analysis indicated that the interaction between the oxygen vacancies on the catalyst surface and the active sites of multiple metals was the main pathway for catalytic reactions. This study provides a beneficial attempt for the methanation of fermentation gas and biogas. This study provides a beneficial attempt for the methanation of bioethanol fermentation gas and biogas.

## Data Availability

The authors will supply the relevant data in response to reasonable requests.
